# Enhanced Photostability and Photoactivity of Ruthenium Polypyridyl-Based Photocatalysts by Covalently Anchoring Onto Reduced Graphene Oxide

**DOI:** 10.1021/acsomega.3c08800

**Published:** 2024-03-14

**Authors:** Seán Hennessey, Roberto González-Gómez, Kathryn McCarthy, Christopher S. Burke, Camille Le Houérou, Nirod Kumar Sarangi, Patrick McArdle, Tia E. Keyes, Fabio Cucinotta, Pau Farràs

**Affiliations:** † School of Biological and Chemical Sciences, Energy Research Centre, Ryan Institute, University of Galway, H91 CF50 Galway, Ireland; ‡ School of Chemical Sciences, National Centre for Sensor Research, Dublin City University, Dublin 9, Ireland; § School of Chemistry and Analytical and Biological Chemistry Research Facility (ABCRF), University College Cork, T12 K8AF Cork, Ireland; ∥ School of Natural and Environmental Sciences, Bedson Building, Newcastle University, Newcastle upon Tyne NE1 7RU, U.K.

## Abstract

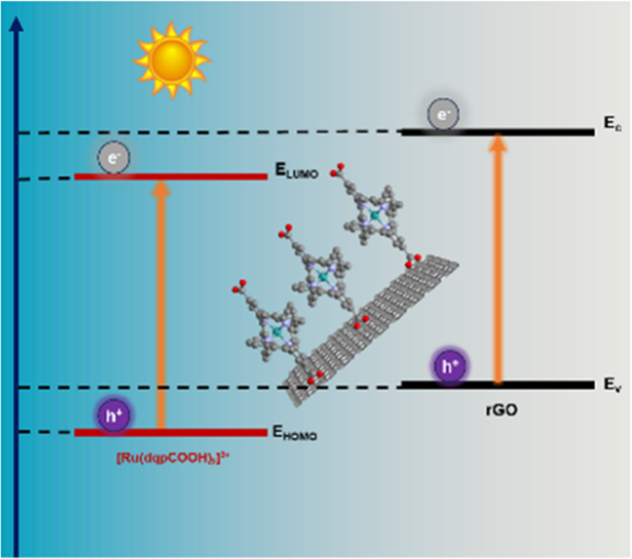

Recentstudies toward finding more efficient ruthenium metalloligands for photocatalysis applications have shown that the derivatives of the linear [Ru(dqp)_2_]^2+^ (dqp: 2,6-di(quinolin-8-yl)-pyridine) complexes hold significant promise due to their extended emission lifetime in the μs time scale while retaining comparable redox potential, extinction coefficients, and absorption profile in the visible region to [Ru(bpy)_3_]^2+^ (bpy: 2,2′-bipyridine) and [Ru(tpy)_2_]^2+^ (tpy: 2,2′:6′,2″-terpyridine) complexes. Nevertheless, its photostability in aqueous solution needs to be improved for its widespread use in photocatalysis. Carbon-based supports have arisen as potential solutions for improving photostability and photocatalytic activity, yet their effect greatly depends on the interaction of the metal complex with the support. Herein, we present a strategy for obtaining Ru–polypyridyl complexes covalently linked to aminated reduced graphene oxide (rGO) to generate novel materials with long-term photostability and increased photoactivity. Specifically, the hybrid Ru(dqp)@rGO system has shown excellent photostable behavior during 24 h of continual irradiation, with an enhancement of 10 and 15% of photocatalytic dye degradation in comparison with [Ru(dqp)_2_]^2+^ and Ru(tpy)@rGO, respectively, as well as remarkable recyclability. The presented strategy corroborates the potential of [Ru(dqp)_2_]^2+^ as an interesting photoactive molecule to produce more advantageous light-active materials by covalent attachment onto carbon-based supports.

## 1 Introduction

The natural processes involved in photosynthesis have inspired researchers to develop cleaner and more sustainable methods to produce value-added chemicals from small molecules.^1^ In many cases, this has led to the synthesis of highly efficient molecular photocatalysts for use in a wide array of chemical transformations, namely, [Ru(bpy)_3_]^2+^ and [Ir(ppy)_3_]^2+^ (ppy: 2-phenylpyridine). However, the irrecoverable nature, irretrievable losses, product recovery issues, and risk of product contamination of catalysts reduce their window of opportunity and attractiveness for photocatalysis.^2^ Consequently, hybrid assemblies based on light-responsive molecules have been designed for the fabrication of light-active materials.^3^ Their utilization in frameworks or incorporation into conductive supports, such as graphene-based systems, has led to a vast array of hybrid systems, promoting the use of solar irradiation to drive chemical reactions.^4−7^ To promising effect, light-active hybrid materials have also been utilized for redox chemical transformations that could not be efficiently achieved by conventional chemical synthesis methods, highlighting the potential of these materials in the field of sustainable catalysis.^8^

Recently, a plethora of hybrid systems ranging from metal oxides and nanoparticles to silicon and carbon-based materials have been used to improve the stability of light-active molecules and enhance their performance in photocatalytic transformations.^9−11^ Of particular interest is the incorporation of photoresponsive molecular systems into rGO, which has been shown to be an effective and reliable conductive support due to its tunable electronic properties and potential for high scalability.^12,13^

The variety of molecular complexes that have been used in both photosensitizing and photocatalysis is extensive, with special attention to the diverse class of highly active polypyridyl-based metal complexes.^14−17^ Due to their well-known photophysical properties, ruthenium polypyridyl complexes have been widely used in photobased applications for many years,^18^ with [Ru(bpy)_3_]^2+^-based molecules being the most extensively studied, through both covalent interactions, as well as adsorption on photoactive surfaces.^19−22^ Yet, conventional Ru^2+^ polypyridyl complexes, under some conditions, can show photochemical instability due to their low-lying triplet metal-centered states (e_g_), and when incorporated into materials, this phenomenon persists.^23^ Bis homoleptic ruthenium polypyridyl complexes with tridentate ligands such as [Ru(tpy)_2_]^2+^ and [Ru(dqp)_2_]^2+^ are systems of interest because of their achiral and linear nature, aiding in architectural control of supramolecular assemblies and interfacial systems; however, they have been relatively overlooked in photocatalytic systems, especially that of [Ru(dqp)_2_]^2+^. Other heteroleptic ruthenium complexes have been covalently linked to a COCl-GO support and employed for photocatalytic CO_2_ reduction,^24^ while several examples use a noncovalent strategy using π–π stacking of a pyrene-functionalized Ru complex for photocatalysis and photoredox catalysis in aqueous solutions.^25,26^

[Ru(tpy)_2_]^2+^ complexes likely because the strain induced by the bite angle of the tridentate ligand promotes radiationless decay and population of ^3^MC states leading to a comparatively very short lifetime of their excited state, limiting their potential and applicability in photocatalysis.^27^ In this regard, the recent incorporation of [Ru(tpy)_2_]^2+^ complexes into photoactive materials has shown promising enhancement of the hybrid materials’ photocatalytic performance.^26,28−30^ Wilhelm and co-workers showed significantly improved photocatalytic activity when anchoring a [Ru(tpy)_2_]^2+^ complex onto graphene oxide (GO).^31^

By opening the N–Ru–N bite angles of the coordination cage in the tridentate ligand, the photophysical properties can be dramatically improved, including most notably the excited state lifetime, while retaining the geometric advantages of the [Ru(tpy)_2_]^2+^ complexes.^32−36^ The family of [Ru(dqp)_2_]^2+^ complexes have been used in photoredox-active films in the form of metallopolymers formed via electropolymerization,^37,38^ nevertheless, even when presenting comparable properties than [Ru(bpy)_2_]^2+^ and [Ru(tpy)_2_]^2+^ complexes, in terms of absorption profile in the visible region and extinction coefficients, its application in photoresponsive materials can be considered as underexplored.^39^ Recent work in our research group has shown how the incorporation of a [Ru(dqp)_2_]^2+^ species into a photoactive metallopolymer exhibits exceptional photostability under intense solar irradiation, opening potential avenues into the use of these metal complexes in hybrid materials for their application in highly stable photocatalysis.^40^

**Figure 1:**
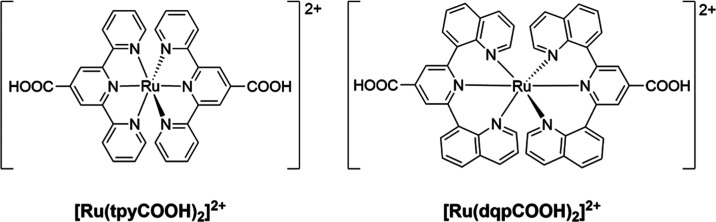
Metal complexes studied in this work.

A series of electro- and photocatalytic transformations have been performed previously with [Ru(bpy)_3_]^2+^ derivatives covalently anchored on rGO for CO_2_ reduction,^41^ water-splitting,^42,43^ organic reactions,^44^ and light-harvesting,^45,46^ highlighting the importance of the covalent linkage in the hybrid materials. Accordingly, we focus on the elaboration of a modified ruthenium polypyridyl complex with carboxylic acids as pending groups, namely, [Ru(dqpCOOH)_2_]^2+^, whose crystal structure is unveiled in this work. The light-active molecule was covalently anchored onto aminated rGO to enhance its photostability and improve its performance in photocatalytic reactions. The photocatalytic performance of the new hybrid material, Ru(dqp)@rGO, was compared to its free molecular counterpart and to both modified [Ru(tpy)_2_]^2+^ molecular complex and its anchored hybrid analogue, namely, [Ru(tpyCOOH)_2_]^2+^ and Ru(tpy)@rGO, respectively (Figure 1). The new material has shown outstanding photostability and noteworthy performance in the degradation of the model organic pollutant methylene blue (MB), as well as significant recyclability over three cycles.

## 2 Results and Discussion

### 2.1 Material Fabrication

**Figure 2:**
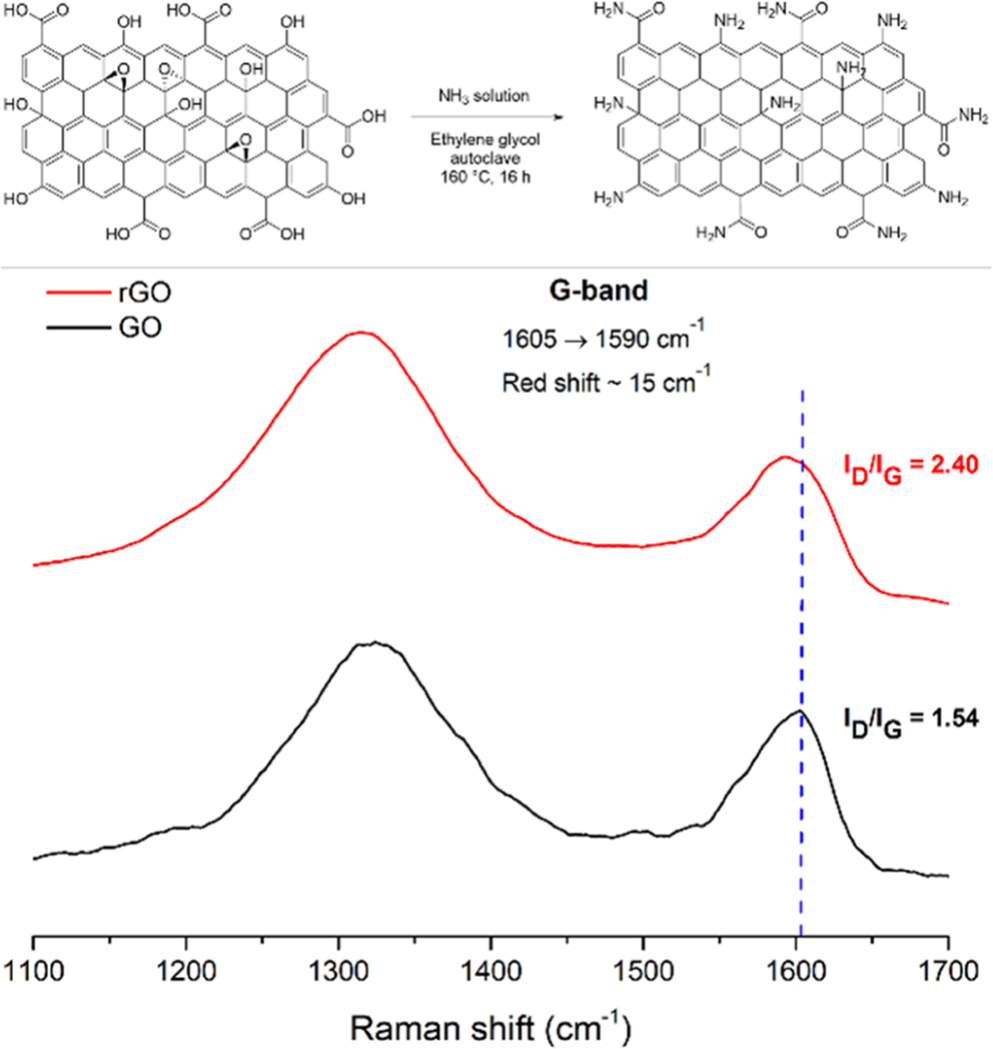
Synthesis route of the reduction of GO to produce rGO (top). Raman spectra of GO (black) and rGO (red), emphasizing the red shift of the G-band. The ratio in intensity between the D (ca. 1350 cm^–1^) and G bands (*I*_D_/*I*_G_) are also highlighted (bottom).

The synthetic procedure for the preparation of the aminated rGO (from here on termed rGO) is described in detail in the Section 4. By following a modified literature methodology, commercial graphene oxide (GO) was reduced and subsequently aminated in a one-pot autoclave procedure using ethylene glycol and ammonia at ca. 155 °C.^45^ Raman spectroscopy confirmed the formation of the rGO, which displays a red shift in the G-band of the material, ca. 15 cm^–1^, due to the oxidation of the graphene net, as well as significant changes in the intensity ratio of the D and G bands (*I*_D_/*I*_G_), in comparison with GO (Figure 2).^47^ In addition, powder X-ray diffraction (PXRD), thermogravimetric analysis (TGA), and solid-state ultraviolet–visible spectroscopy (ss-UV–vis) of the synthesized material display similar results to literature examples of rGO (see Supporting Information).^47,48^

For the synthesis of the two ruthenium complexes [Ru(tpyCOOH)_2_]^2+^ (**1**) and [Ru(dqpCOOH)_2_]^2+^ (**2**), literature procedures were followed.^40,49^ The unknown crystal structure of **2**, reported here for the first time, was determined using single crystals isolated by hot crystallization from a DMF/H_2_O mixture (Figure S1, Table S2). The crystal structure was solved using ShelxT^50^ and refined using ShelxL^51^ both of which were operated using the Oscail package.^52^ The crystal structure contains a Ru^2+^ octahedra with two tridentate ligands and an oxidation state of −1; the deprotonated carboxylic acids are linked by hydrogen-bonded water molecules, the hydrogen atoms of which were also located and refined.

The complexes were first activated by converting the carboxylic pending groups to acetyl chlorides, which was corroborated by IR (Figure S2). The modified complexes were then transferred to a predispersed suspension of rGO containing triethylamine in chloroform and further sonicated for 3 h. The solid dispersion was isolated by filtration, washed with acetonitrile and water, and then oven-dried to give the ruthenium-anchored rGO (Ru(L)@rGO, where L = tpy or dqp).

### 2.2 Material Characterization

**Figure 3:**
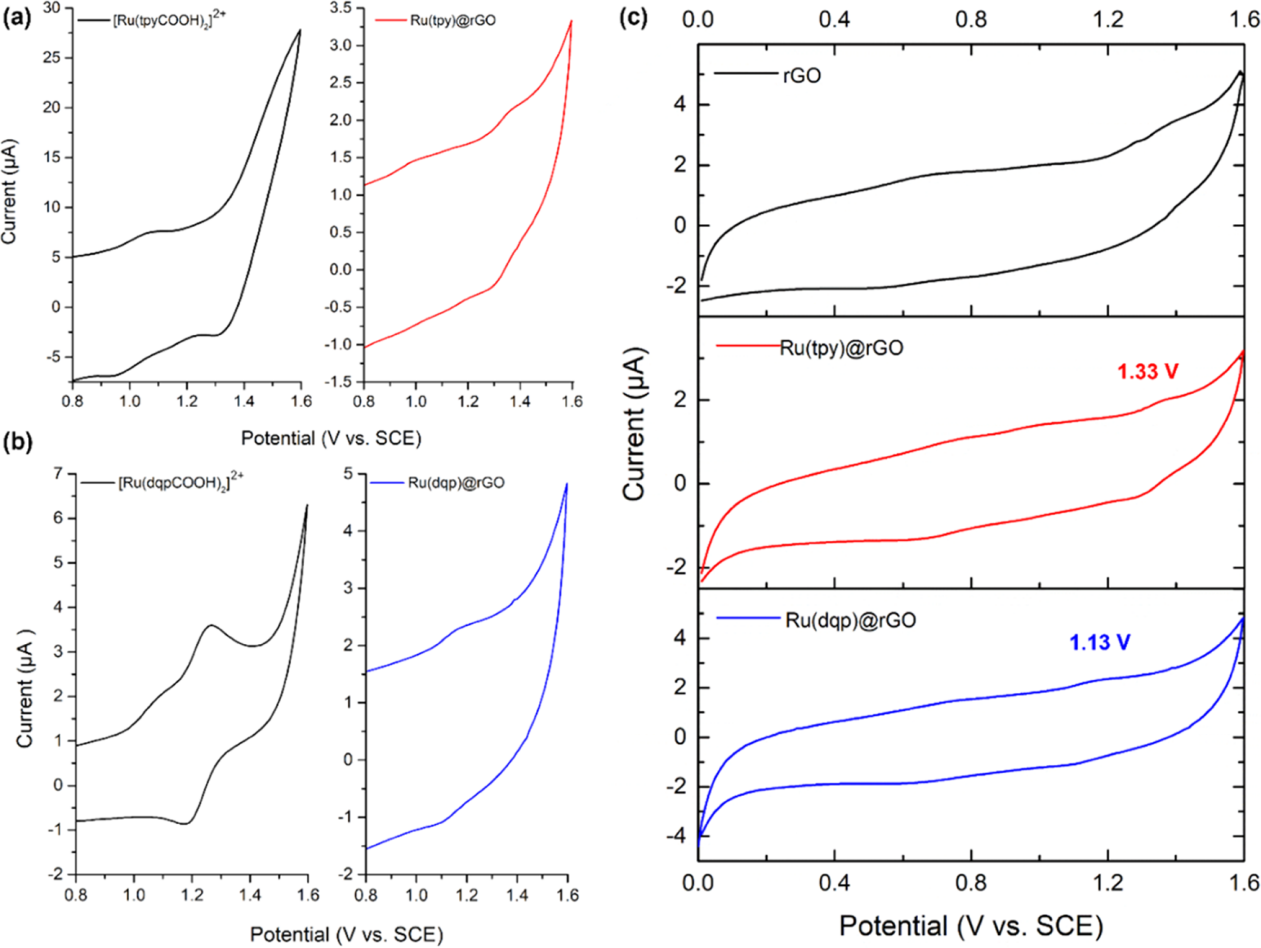
Cyclic voltammetry comparisons of: (a) the [Ru(tpyCOOH)_2_]^2+^ complex (black) and the Ru(tpy)@rGO material (red). (b) [Ru(dqpCOOH)_2_]^2+^ molecule (black) and the Ru(dqp)@rGO system (blue). (c) rGO support (black), Ru(tpy)@rGO hybrid material (red), and Ru(dqp)@rGO hybrid system (blue). All measurements were performed in N_2_-bubbled CH_3_CN at a scan rate of 0.1 V s^–1^. rGO-based materials were drop cast onto glassy carbon electrodes via an EtOH/Nafion suspension.

The covalent attachment of the complex to rGO was first confirmed via electrochemistry and by comparing the properties of the Ru(L)rGO materials with their free complex counterparts (Figure 3a,b). Due to the low solubility and nondispersible nature of the synthesized materials, the rGO hybrid systems were challenging to measure quantitatively, reflected in the scarcity of electrochemical characterizations performed in the literature. To overcome this issue, a small quantity of the rGO hybrid material was sonicated in a solution of EtOH/Nafion for 3 h before being drop cast onto a glassy carbon (GC) electrode and dried overnight. The methodology allowed the building of a working electrode in which the Ru^III/II^ couple could be reliably observed. In both cases, the presence of an albeit weak voltammetric signal yielded *E*_1/2_ values corresponding to the Ru^III/II^ reversible couple at +1.33 and +1.13 V_SCE_ for Ru(tpy)@rGO and Ru(dqp)@rGO, respectively. Interestingly, the reversible couple for the free [Ru(tpyCOOH)_2_]^2+^ complex at +1.15 V_SCE_ is shifted much more significantly (+1.08 → + 1.33 V_SCE_) in comparison to that of [Ru(dqpCOOH)_2_]^2+^ (+1.22 → + 1.13 V_SCE_). Compared to the bare rGO, where no oxidation or reduction peaks were observed, the redox reversible peaks corresponding to the Ru^III/II^ couple can clearly be distinguished in both materials, confirming that the Ru complexes were successfully covalently linked (Figures 3c and S3).

As infrared spectra of rGO-anchored complexes are known to be difficult to measure due to the thickness of the sample,^53^ Raman spectra at 785 nm were performed to further ascertain the anchoring of the [Ru(tpyCOOH)_2_] complex onto the rGO surface (Figure S4). Signals at 420, 820, 840, and 1780 cm^–1^, respectively, correspond with similar ruthenium complex values, with the main expected signals between 1200 and 1600 cm^–1^ overshadowed by that of the rGO signals.^54^ However, due to the lower loading of [Ru(dqpCOOH)_2_], it was not possible to see strong signals in its corresponding Raman spectra.

Furthermore, scanning electron microscopy with energy-dispersive X-ray spectroscopy (SEM-EDX) was used to further characterize the material and reinforce the findings in electrochemistry (Figures S5–S7). Hybrid materials displayed no morphology evolution regarding the bare rGO, as observed by SEM; meanwhile, EDX confirmed the presence of the Ru complexes throughout the material. The loading percentage of Ru complex into each hybrid material was determined by EDX measurements. The percentage of Ru/C mass normalized ratio for the Ru(tpy)@rGO was found to be 5.47%, whereas in the case of Ru(dqp)@rGO, the anchoring percentage dropped to 1.34%.

The thermal stability of the materials was determined by TGA (Figure S8), with both Ru-anchored materials exhibiting slightly higher thermal stabilities at >500 °C compared to the bare rGO, in line with other rGO-based composites reported in the literature.^55−57^ Furthermore, PXRDs of the hybrid materials and bare rGO presented consistently two main peaks at ca. 27 and 43°, attributed to the (002) and (101) reflections. The appearance of new or shifted signals was not detected, suggesting no apparent effects on the rGO by loading the Ru complexes, in terms of lattice parameters, leaving the *d*-spacing between the rGO layers unmodified (Figure S9).^43^

### 2.3 Photophysics and Photostability

**Figure 4:**
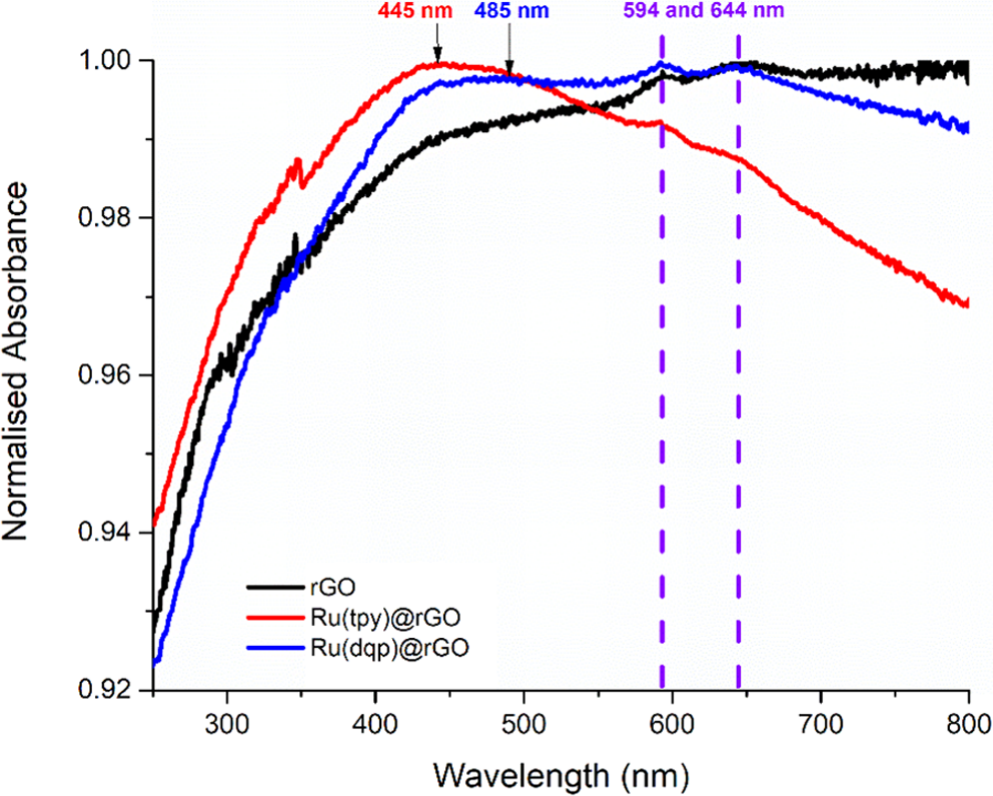
ss-UV–vis spectra comparisons of rGO (black), Ru(tpy)@rGO (red), and Ru(dqp)@rGO (blue). The MLCT transitions for each anchored complex are highlighted, as well as the bands corresponding to the π-conjugated structure (dashed purple).

The hybrid materials were characterized initially by ss-UV–vis spectroscopy to investigate their optical properties and, afterward, by photoluminescence spectroscopy to determine the excited state lifetime and long-term photostability of the Ru complex in the hybrid systems. The absorption profile of the materials obtained by ss-UV–vis (Figure 4) presents distinctive bands at 594 and 644 nm for both hybrid materials and rGO, which is attributed to the low-modified π-conjugated structure of the rGO. Meanwhile, the spectrum of the carbon-based support exhibits a shoulder-type band at 290 nm, which is assigned to the red-shifted (in comparison to bare GO) π–π* transition in the rGO material.^47^ Regarding the two Ru-anchored materials, bands in the ss-UV–vis spectra at ca. 440–460 and 475–495 nm were distinguished for Ru(tpy)@rGO and Ru(dqp)@rGO, respectively. The appearance of these two bands is attributed to the metal–ligand charge transfer (MLCT) transitions of each complex, which typically appear in the 440–600 nm region (Figure S10).

**Figure 5:**
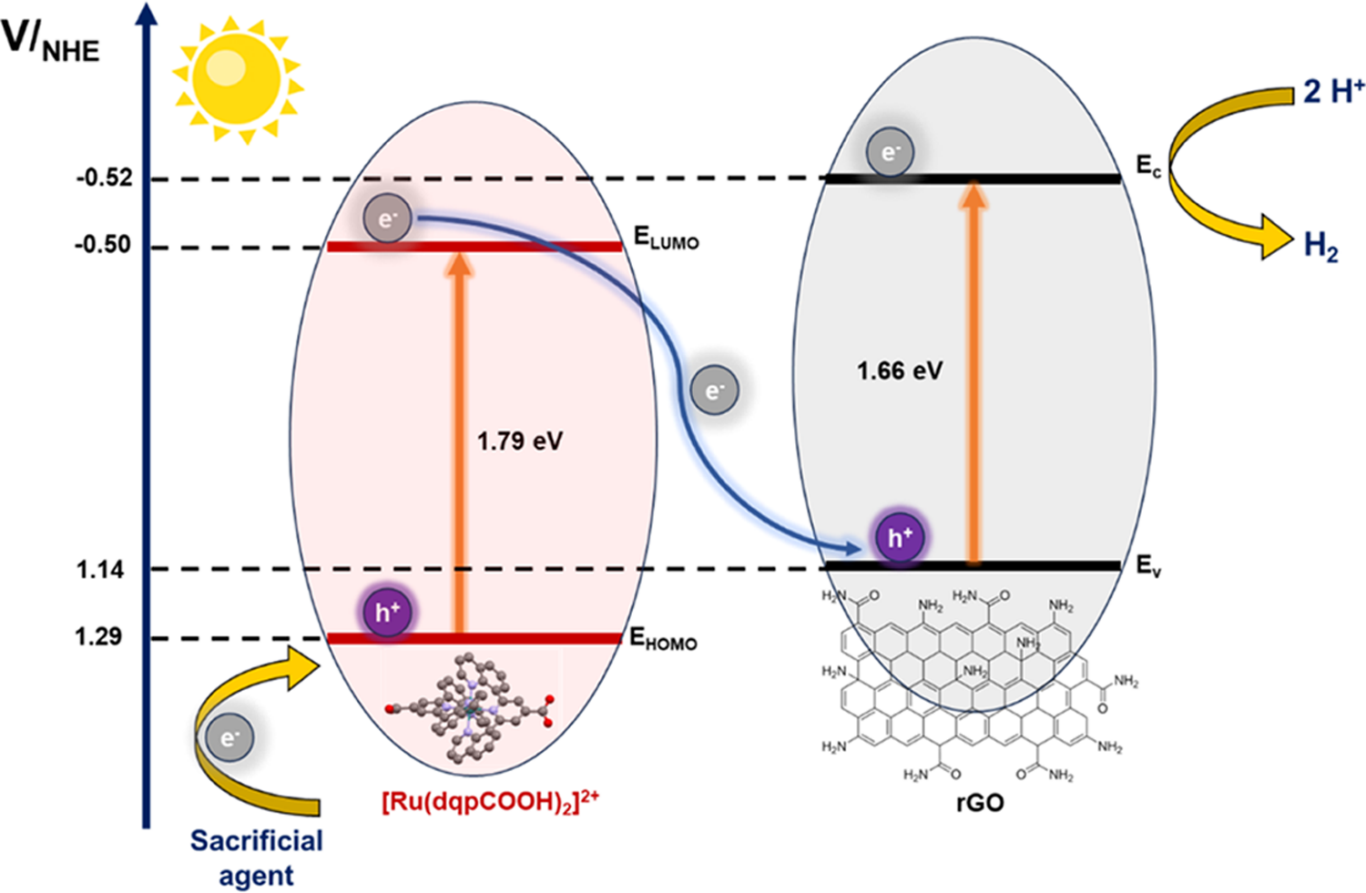
Schematic representation of the possible *Z*-scheme mechanism for the movement of electrons as a result of photogenerated holes in Ru(dqp)@rGO.

Experimental data obtained from ss-UV–vis and electrochemistry of the Ru[(dqpCOOH)_2_]^2+^ free complex and rGO support were utilized to predict the energy level diagram of the Ru(dqp)@rGO hybrid material (Figure 5). A 1.66 eV band gap of the rGO was calculated by estimation from the Tauc plot calculated from its absorbance spectrum (Figure S11a), which is higher than that of typical rGO in the literature, which is attributed to the degree of substitution in the reduced surface.^58^ Meanwhile, the conduction band potential of rGO was established as −0.52 V_NHE_ at pH = 0, as described in previous work by Gong et al.^59^ On the other hand, the highest occupied molecular orbital (HOMO) and lowest unoccupied molecular orbital (LUMO) energy value of the [Ru(dqpCOOH)_2_]^2+^ complex was estimated from cyclic voltammetry (CV) studies and absorbance spectra, respectively (Figures 3b and S11b), showing a band gap of 1.79 eV. The HOMO and LUMO levels of the [Ru(dqpCOOH)_2_]^2+^ and rGO were calculated to be −0.50 and +1.14 eV, respectively. Under visible light conditions, an electron–hole pair is generated from the Ru complex anchored on the rGO surface. As seen in Figure 5, the band alignment between both units indicates that the transfer of photogenerated electrons to the rGO surface is thermodynamically favored, whereupon adsorbed oxygen (O_2_) can form superoxide radicals (^•^O_2_^–^), which are used in organic pollutant photodegradation processes.^60,61^

**Figure 6:**
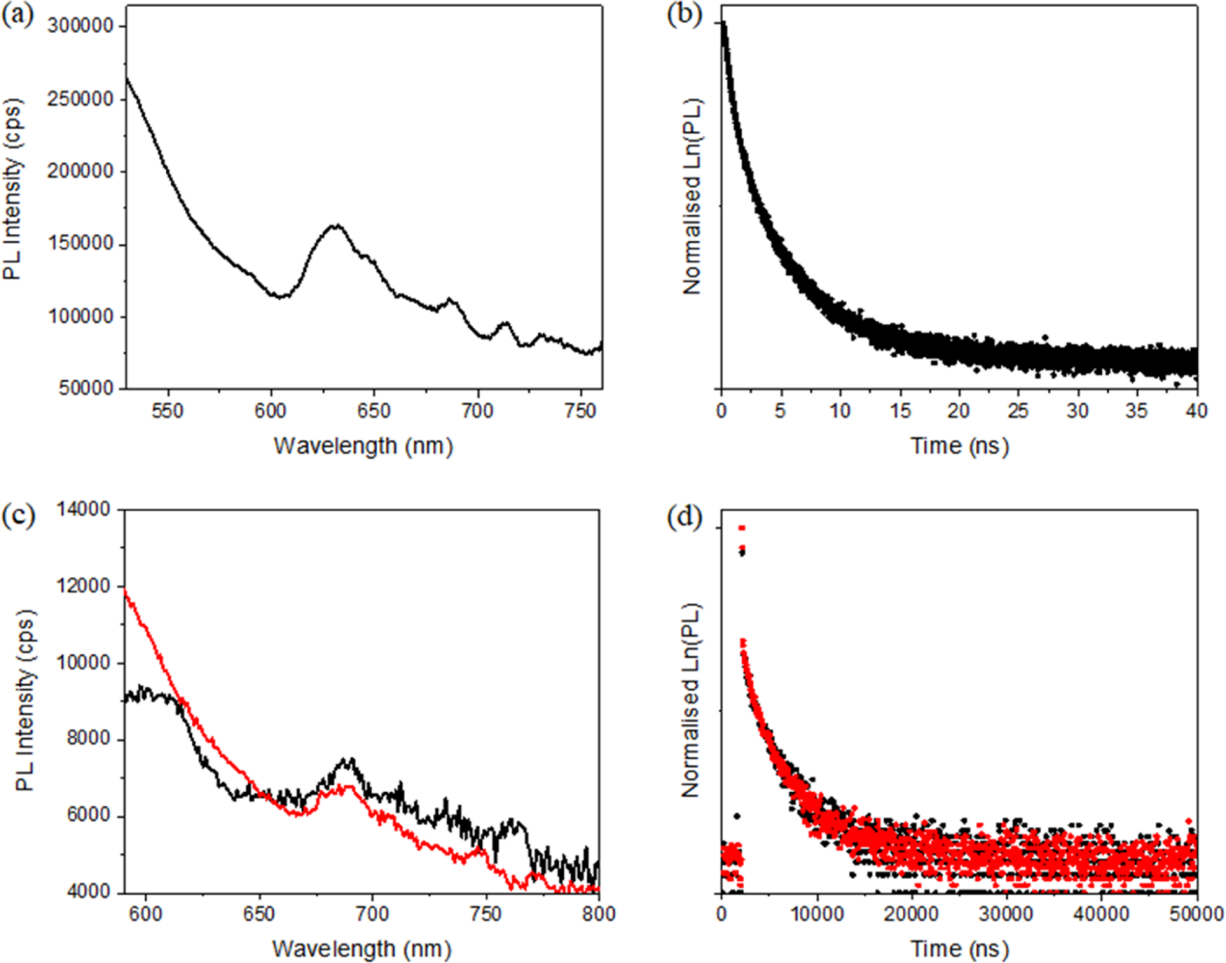
(a): Emission spectrum of Ru(tpy)@rGO, recorded in the solid state at λ_exc_ = 300 nm, with 10 nm slits. (b): excited-state decay profile of Ru(tpy)@rGO, recorded in the solid state at λ_em_ = 650 nm; the fitted traces are 0.92 and 4.90 ns with a relative weight of 40 and 60%, respectively, and an average of 3.32 ns. (c): emission spectrum of Ru(dqp)@rGO, recorded in the solid state at λ_exc_ = 480 nm, with 10 nm slits, before (black) and after (red) irradiation for 24 h. (d): excited-state decay profile of Ru(dqp)@rGO, recorded at λ_em_ = 690 nm, before (black) and after irradiation (red).

Concerning photoluminescence spectroscopy, despite the weak emission of the Ru(tpy)@rGO hybrid material, it was possible to record an excited-state decay profile (Figure 6a,b). The obtained data were fitted to a biexponential profile, giving an average lifetime of 3.32 ns. Since the excited-state lifetime of the [Ru(tpyCOOH)_2_]^2+^ complex in deaerated acetonitrile is 25.6 ns,^62^ our measurements highly suggest a quenching process due to the presence of rGO. In regard to the Ru(dqp)@rGO hybrid system, an even weaker emission band was observed, yet it also pointed out a biexponential excited-state decay profile (Figure 6c). The fitted decay curve yielded values of 6.54 and 456 ns with a relative weight of 54 and 46%, respectively. Overall, the average lifetime calculated is 213 ns, a value shorter than that of the [Ru(dqpCOOH)_2_]^2+^ complex in both deaerated (2.55 μs) and air-equilibrated acetonitrile (314 ns), also denoting the quenching process.^29^

Interestingly, the long component recorded on rGO appears more extended than that in air-equilibrated solution, which might reflect the relatively high rigidity of the ruthenium complexes once grafted on the rGO surface. In addition, the appearance of two decay components could be an indication of different populations of Ru^2+^ complex, which present different sensitivity toward the quenching of rGO. To investigate the effect of covalently anchoring the complex onto rGO in terms of photostability, the hybrid materials were exposed to light for a prolonged period to record any changes in their luminescence properties. Photoirradiation studies were carried out only for the Ru(dqp)@rGO material since the Ru(tpy)@rGO sample presents a relatively quick quenching process, as discussed previously.

A white flood light source was used over the course of 24 h, with no significant changes observed in the emission intensity of the Ru complex (Figures 6c and S12a). Since its emission was very weak even before the irradiation tests, it may not be deemed a reliable parameter to monitor. Therefore, the excited-state decay profile was also recorded after photoirradiation (Figures 6d and S12b), and it proved unaltered, giving strong indications concerning the good photostability of the Ru(dqp)@rGO material.

### 2.4 Photocatalytic Dye Degradation

**Scheme 1:**
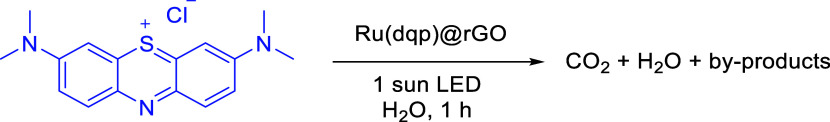
Operational Parameters used for the Photocatalytic Degradation of MB Dye Solution

The photocatalytic properties of the hybrid materials were investigated by the photodecomposition of a well-known organic pollutant, namely, methylene blue (MB) (Scheme 1). Aqueous solutions of MB (10 ppm, 5 mL) were constantly irradiated for 1 h under 1 sun LED illumination (λ > 300 nm, 100 mW cm^–2^) to study the dye photodegradation ratio in the presence of the hybrid materials (2.5 mg). However, to determine the real effects of covalently anchoring the Ru complex onto rGO, MB solutions were exposed to light irradiation in the presence of both bare rGO (2.5 mg) and free Ru complexes (1.0% mol) under the same conditions. After one hour, aliquots were withdrawn from the reaction mixture, and their UV–vis was measured.

**Figure 7:**
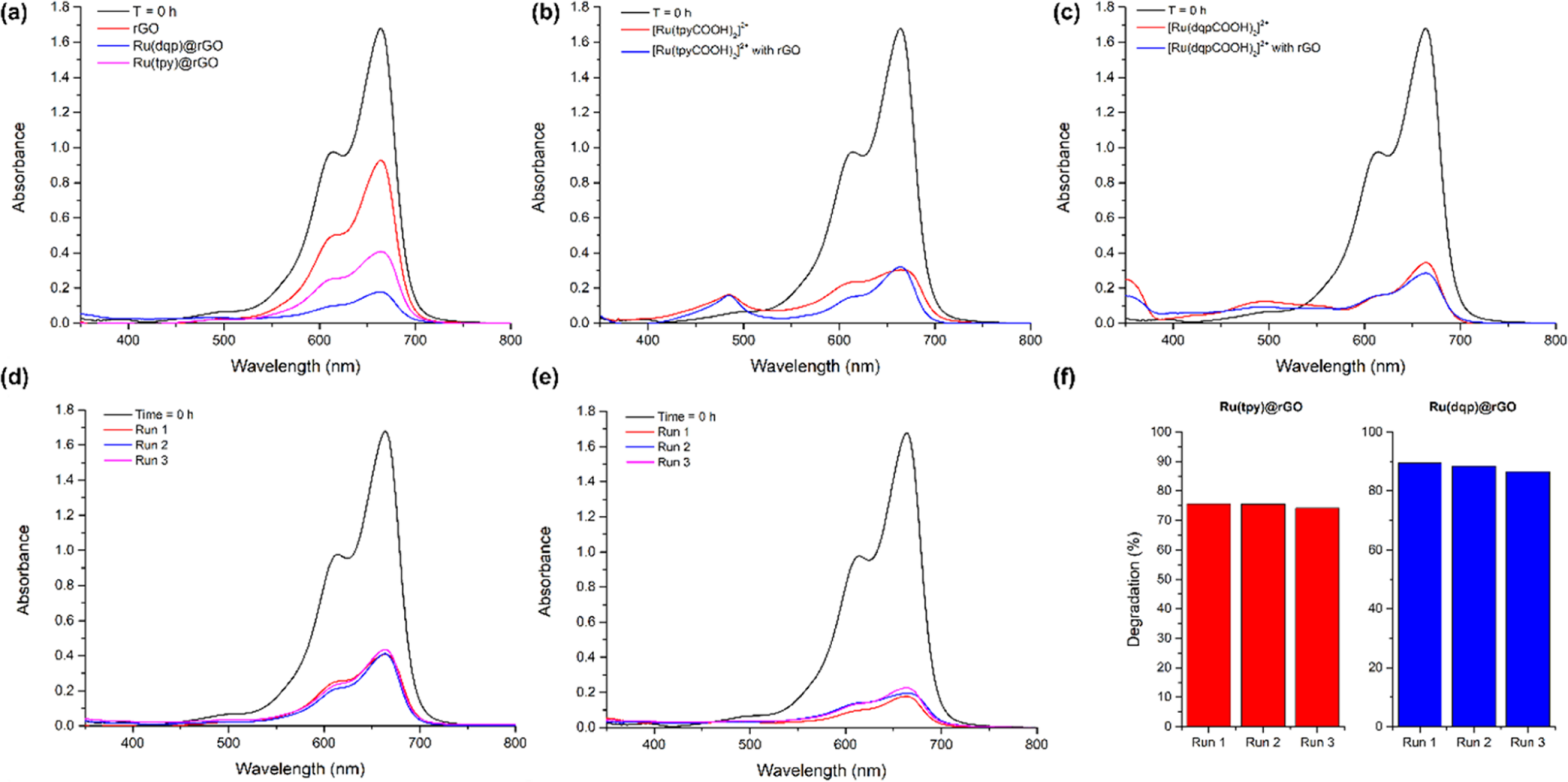
UV–vis absorbance measurements of MB photodegradation experiments after 1 h of irradiation under 1 sun LED. MB absorbance at *t* = 0 h is shown in black in all spectra. (a) bare rGO (red), Ru(tpy)@rGO (blue), and Ru(dqp)@rGO (pink). (b) [Ru(tpyCOOH)_2_]^2+^ (red), and [Ru(tpyCOOH)_2_]^2+^ with rGO (blue). (c) [Ru(dqpCOOH)_2_]^2+^ (red), and [Ru(dqpCOOH)_2_]^2+^ with rGO (blue). (d–f) Comparisons of photodegradation recyclability of Ru(tpy)@rGO and Ru(dqp)@rGO.

Prior to MB irradiation in the presence of the materials, the suspensions were magnetically stirred in the dark for 1 h to ensure an adsorption–desorption equilibrium. The UV–vis of the nonirradiated samples was then measured, determining a ca. 2.4% adsorption of MB into the materials, the absorption of which was taken as *t* = 0 h (Figure 7). After the adsorption–desorption equilibrium was reached, the dye solutions were irradiated under constant stirring. Bare rGO photodegraded ca. 44.8% of the MB; nevertheless, only a slight increase against the dye decomposition (41.6%) without any catalyst under the same light source irradiation was registered. Meanwhile, bare rGO mixed with the ruthenium complexes displayed a much higher effect than only bare rGO, yet slight changes were detected in comparison with the Ru complex. An increase of over 4% versus the molecular catalysts was observed in the case of [Ru(dqpCOOH)_2_]^2+^ mixed with bare rGO, but strangely the opposite was registered for the [Ru(tpyCOOH)_2_]^2+^ system.

Encouragingly, both Ru-anchored materials showed moderate to excellent MB photodegradation after 1 h of irradiation (Table 1, Figure 7d,e). Further to this, no leaching was observed in both of the Ru complex anchored materials, as was shown by the absence of the metalloligand signals in the UV–vis spectra performed after catalysis. The Ru(tpy)@rGO presented a reasonable MB photodegradation of 75.8% after one h of irradiation, less than the analogue complex with the rGO and even less than the free complex in solution. Most promisingly, [Ru(dqpCOOH)_2_]^2+^, when anchored on rGO, showed an MB photodegradation of 89.1%, a remarked improvement of ca. 10.4% over the free Ru complex in solution, which has been shown to degrade rapidly under intense illumination.^40^ It is theorized that due to the extended excited state lifetime of the dqp over that of the tpy, the pathway to degradation of the MB is more readily accessed. SEM images displayed no morphological evolution of the materials after catalysis, as well as nondetectable degradation (Figure S13), emphasizing the good stability of the hybrid systems.

**Table 1:** Photocatalytic Degradation Results of MB

		degradation [%]		
sample	catalyst	run 1	run 2	run 3
**1**		41.6		
**2**	[Ru(tpyCOOH)_2_]^2+^^a^	85.5		
**3**	[Ru(dqpCOOH)_2_]^2+^^a^	79.1		
**4**	rGO^b^	44.8		
**5**	[Ru(tpyCOOH)_2_]^2+^ + rGO^a^^,^^b^	82.1		
**6**	[Ru(dqpCOOH)_2_]^2+^ + rGO^a^^,^^b^	83.0		
**7**	Ru(tpy)@rGO^b^	75.8	75.5	74.2
**8**	Ru(dqp)@rGO^b^	89.5	88.4	86.5

a Reaction conditions: 10 ppm solution of MB in 5 mL distilled water, 1 sun LED illumination (λ > 300 nm, 100 mW cm^–2^), catalyst: 1.0% mol in solution.

b 2.5 mg dispersed.

In order to discern the MB photodegradation mechanism in the presence of the Ru(dqp)@rGO hybrid system, proton nuclear magnetic resonance (^1^H NMR) studies were carried out on a highly concentrated sample of MB. Dye degradation monitoring was performed on an NMR tube containing a 2000 ppm solution of MB in D_2_O and the Ru(dqp)@rGO material, and the suspension was continuously irradiated for 3 h in the same conditions above-mentioned. ^1^H NMR spectra were taken at 30 min intervals to track the degradation process. Upon light interaction, aromatic proton signals of MB, ca. 7.28, 7.00, and 6.78 ppm, underwent a deshielding phenomenon, and a gradual broadening was also observed. Also, the intensities of the peaks were reduced during the monitoring, suggesting a continuous decrease in MB concentration during the experiment (Figure S14).

Greater NMR resolution was achieved by increasing the acquisition time as well as the number of scans. After final irradiation, new peaks at ca. 2.65, 2.51, and 2.00 ppm were distinguished, implying the formation of small aliphatic hydrocarbons as side-products of the MB photodegradation (Figure S15). The observations found during this test agree well with a previous study performed with a similar carbon-based catalyst.^63^ Further evidence and quantification of the MB degradation were obtained using UV–vis, observing a decrease of ca. 7.0% of the initial dye concentration under the same conditions as previously described (Figure S16). Hence, it is theorized that after irradiation, the photoexcited electrons of the rGO produce reactive oxygen species, which combine with water to generate hydroxyl radicals to initiate the MB photodegradation.

## 3 Conclusions

Two Ru–polypyridyl complexes, [Ru(tpyCOOH)_2_]^2+^ and [Ru(dqpCOOH)_2_]^2+^, in conjunction with rGO, have produced hybrid systems with the highly stable photocatalytic degradation of a model organic pollutant. The systems have been characterized extensively using Raman, IR, TGA, and PXRD measurements to confirm that the rGO structure is maintained after chemical modification with the Ru complexes. In addition, using drop casting of Nafion/EtOH solutions, cyclic voltammograms of Ru complexes immobilized on graphene oxide have been obtained for the first time. ss-UV–vis profiles of the materials have also been measured, with clear differences in the absorption spectra being attributed to the MLCT of the Ru complexes. Most promisingly, the Ru(dqp)@rGO material exhibits excellent photostability, as shown by an unchanged emission profile over the course of 24 h of white light irradiation, whereas the Ru(tpy)@rGO displays rather a poor emission and a very short-lived excited state. In the degradation of MB, both rGO-anchored materials exhibited excellent photocatalytic degradation, with only a 2–3% drop-in activity noted in recyclability studies. Overall, we have shown how the Ru(dqp)@rGO hybrid material is more efficient than the Ru(tpy)@rGO system, thereby showcasing the excellent potential of the linear bistridentate complex with reduced bite angles, [Ru(dqp)_2_]^2+^, in both photoactive and photocatalytic applications.

## 4 Experimental Methods

### 4.1 Metalloligand and Material Preparations

*Ruthenium complexes:* Literature procedures were used for the synthesis of [Ru(tpyCOOH)_2_](PF_6_)_2_ and [Ru(dqpCOOH)_2_](PF_6_)_2_.^40,49^^1^H NMR spectra were recorded at 400 or 500 MHz as indicated using Bruker spectrometers and were processed using Bruker Topspin software with calibration against solvent peaks according to published values. Crystals of [Ru(dqpCOOH)_2_]^2+^ were developed by the dissolution of 5 mg of the complex in a 3 mL solution of DMF/H_2_O (3:2) and heating to 100 °C. The solution was then cooled to room temperature to give dark red crystals, and the obtained crystals were kept in their mother liquor before single-crystal diffraction measurements. *Aminated graphene oxide (rGO)*: A modified literature procedure was used as inspiration for this synthesis.^64^ To a 100 mL Teflon vessel was added 7 mL of graphene oxide (0.4% w/v), 2 mL of NH_3_ (35% solution), and 55 mL of ethylene glycol. The mixture was stirred at room temperature for 5 min before being transferred to a stainless-steel autoclave and placed in a preheated oven at 155 °C for 20 h. Upon cooling, the mixture was filtered, and the black precipitate was washed with copious amounts of water to remove unreacted NH_4_OH and any poly(ethylene glycol) side product. The sample was air-dried via filtration and then placed in an oven at 90 °C for 12 h to give the aminated graphene oxide as a black powder (0.025 g). *Ruthenium anchoring on aminated graphene oxide (Ru(L)@rGO)*: (i) Preparation of chlorocarbonyl tethered ruthenium complex: To a 25 mL round-bottom flask was added the ruthenium complex (0.030 mmol) dissolved in CH_3_CN (10 mL). To the stirring mixture was added SOCl_2_ (0.300 mmol, 5 equiv) and the mixture stirred for 90 min. The solvent was removed under a vacuum to give the chlorocarbonyl tethered ruthenium complex. (ii) *Dispersion of rGO*: To a 10 mL glass vial was added 30 mg of rGO and 10 mL of CHCl_3_. The suspension was sonicated for 1 h at room temperature. To the dispersed rGO was then added NEt_3_ (0.215 mmol, 7.15 equiv) and the suspension was sonicated for a further 5 min. (iii) *Ruthenium Anchoring onto rGO:* To the dispersed suspension of rGO was added the chlorocarbonyl tethered ruthenium complex (0.030 mmol), and the suspension was sonicated for 3 h at room temperature. The reaction vessel was allowed to settle for 12 h, and the precipitate was filtered and washed with CH_3_CN and H_2_O until colorless to give a black powder, which was dried overnight at 90 °C to give the ruthenium-anchored rGO (Ru(L)@rGO) (Where L = tpy or dqp).

### 4.2 Structural Characterizations

A variety of characterization techniques were used to thoroughly prove the anchoring of the complexes and the subsequent properties of the as-prepared materials. Infrared spectroscopy: IRs (4000–650 cm^–1^) were recorded using a PerkinElmer 16PC FT-IR spectrometer with a KBr reference. Raman spectroscopy: Raman spectra of rGO were acquired using a Witec Alpha 500 confocal laser Raman microscope in upright configuration with a 100 μm fiber Toptica 785 nm laser, a 600 grooves/mm diffraction grating, and an Andor Idus CCD camera (Andor Technology Ltd., Belfast, Ireland). The spatial resolution of the laser was approximately 1 μm. The system was calibrated to the standard silicon peak of 520 cm^–1^. Spectral resolution for all measurements was 1.5 cm^–1^. Spectra were treated for cosmic ray removal and processed using background subtraction and Savitzky–Golay smoothing (Witec Project 4 software version 4.1). Spectra of the anchored materials were collected on a LabRAMHR, Horiba spectrometer (HORIBA UK LTD) with a 10× objective lens exciting with a 785 nm diode laser (10 μW). Instrument auto-calibration was performed prior to measurement using the Rayleigh line and crystalline Si peak at 520 cm^–1^ from the silicon wafer. The spectra were collected from solid deposited onto a microscope slide, and replicates were collected over time to ensure that no spectral changes, e.g., from sample burning, were occurring. Thermal gravimetric analysis (TGA): The experiments were performed by Dr. Manuel Ruether of Trinity College Dublin using a Pyris 1 thermogravimetric analyzer. The heating rate was maintained at a constant rate of 20 °C min^–1^, and all runs were carried out between 25 and 600 °C. The measurements were made using open aluminum crucibles with the system purged using nitrogen. Powder X-ray diffraction: Diffraction patterns were measured on a Bruker D2 Phaser instrument operating using Cu Kα (λ = 1.54178 Å) radiation source and a Lynxeye detector at room temperature, with samples mounted on a zero-background silicon single-crystal sample stage. Electrochemistry measurements: Cyclic voltammograms (CV) were performed in N_2_-bubbled dry CH_3_CN with a 0.1 M NH_4_PF_6_ supporting electrolyte at a scan rate of 0.100 V s^–1^. The standard cell used a glassy carbon working electrode, a platinum electrode as a counter electrode, and a standard saturated calomel electrode (SCE) as a reference. A PalmSense-3 potentiostat was used to record the measurements, which were performed at room temperature. For drop-cast measurements, around 1.0 mg of the material was suspended in a 1.0 mL of a mixture of Nafion solution, which was sonicated for 5 min. The suspension was added dropwise onto the glassy carbon electrode and air-dried for 24 h before measurements. Microscopy measurements: All microscopy measurements were performed in the “Center for Microscopy and Imaging” at the University of Galway, Ireland. SEM-EDX measurements were carried out on a Hitachi S-4700 SEM instrument with an EDX spectrometer. Samples were dispersed in absolute EtOH via 30–60 min sonication. The resulting mixtures were then drop-cast and dried on clean silicon wafers (5 mm × 5 mm). The final samples were coated with gold before the SEM measurements. Photophysical measurements: UV–vis spectrometry was carried out on a Cary 5000 UV–vis-NIR spectrometer (200–2500 nm range) with a deuterium UV lamp light source using R928PTM (UV–vis) or polytetrafluoroethylene (diffuse reflectance spectroscopy (DRS)) detectors using a xenon lamp. Liquid samples were diluted to between 10 and 50 μM, and blank samples were measured using 100% solvent before measurements. Samples for photophysical characterizations used 1.0 cm width quartz cuvettes for all measurements. For solid-state measurements, a diffuse reflectance accessory (DRA) was used, with powder samples dried thoroughly before use and pure MgO used as a blank reference. Time-resolved luminescence measurements: Excited-state lifetimes were measured using an Edinburgh FLS980 photoluminescence spectrometer, equipped with a 450 W xenon arc lamp, Czerny Turner excitation and emission monochromators (1.8 nm mm^–1^ dispersion; 1800 grooves mm^–1^), time-correlated single photon counting (TCSPC) module, and a Hamamatsu R928 P photomultiplier tube (in fan assisted TE cooled housing, operating temperature −20 °C). For lifetime measurements, samples were excited with an EPL-375 (370.8 nm; 61.1 ps pulse width) and an EPL-475 (471.8 nm; 61.1 ps pulse width) picosecond pulsed diode lasers, and data analysis was performed on the F980 software with numerical data reconvolution based on Marquardt–Levenberg algorithm.

### 4.3 Photocatalytic Measurements

Photocatalytic tests were performed using a TF-PE300BF solar light simulator at an intensity of 1 sun LED illumination. Samples of MB were made up to a concentration of 10 ppm and stirred in the dark for 1 h with the catalyst of interest before illumination. Then, samples were irradiated for 1 h and covered, and an aliquot was taken, and the UV–vis was measured. For recycling studies, the catalysts were isolated by filtration, washed with copious amounts of water, dried at 60 °C for 3 h, and placed in a fresh solution of MB for the next catalytic run. NMR degradation experiment: Dye degradation studies were monitored using an NMR tube containing a 2000 ppm solution of MB in 0.5 mL of D_2_O and the Ru(dqp)@rGO material. The suspension was irradiated for 3 h in the same conditions as the photocatalytic aforementioned measurements. The NMR was measured at 30 min intervals, covering from the light between measurements.

## Supporting Information Available

Chemicals and reagents used; IR and electrochemical comparisons; SEM-EDX images; TGA and PXRD comparisons; photophysical measurements and postcatalytic NMR and UV–vis data (PDF)
sh1005 (CIF)

## Supplementary Material

ao3c08800_si_001.pdf

ao3c08800_si_002.cif
