# Supplementary material for: Enhanced Photostability and Photoactivity of Ruthenium Polypyridyl-Based Photocatalysts by Covalently Anchoring Onto Reduced Graphene Oxide
Source: ACS Omega. 2024 Mar 14;9(12):13872–82. doi: 10.1021/acsomega.3c08800 (PMC10976380; doi:10.1021/acsomega.3c08800)
Supplement: Supplementary file 1 — ao3c08800_si_001.pdf [file ao3c08800_si_001.pdf]

Supporting Information

Enhanced photostability and photoactivity of ruthenium polypyridyl-based photocatalysts by covalently anchoring onto reduced graphene oxide.

Seán Hennessey,[a] Roberto González-Gómez,[a] Kathryn McCarthy,[a] Christopher S. Burke,[b,c] Camille Le Houérou,[a] Nirod Kumar Sarangi,[b] Patrick McArdle,[a] Tia E. Keyes,[b] Fabio Cucinotta[d] and Pau Farràs[a*]*

a - Dr. Seán Hennessey, Dr. Roberto González-Gómez, Kathryn McCarthy, Camille Le Houérou, Prof. Patrick McArdle and Dr. Pau Farràs

School of Biological and Chemical Sciences, Energy Research Centre, Ryan Institute, University of Galway, H91 CF50 Galway, Ireland.

E-mail: roberto.gonzalez@universityofgalway.ie and pau.farras@universityofgalway.ie

b - Prof. Tia E. Keyes

School of Chemical Sciences, National Centre for Sensor Research, Dublin City University, Dublin 9, Ireland.

c - Dr. Christopher S. Burke

School of Chemistry and Analytical and Biological Chemistry Research Facility (ABCRF), University College Cork, Cork, Ireland.

d - Dr. Fabio Cucinotta

School of Natural and Environmental Sciences, Bedson Building, Newcastle University, Newcastle upon Tyne, UK.

1. Chemicals and Solvents

Chemicals used in this work.

Chemical	Supplier	Purity (%)
2-Acetylpyridine	Sigma-Aldrich	≥99.0
Ammonium hydroxide solution	Sigma-Aldrich	28.0–30.0
1,1′-bis(diphenylphosphino)ferrocene] dichloropalladium(II)-dichloromethane	Sigma-Aldrich	97.0
2,6-dibromopyridine-4-carboxylic acid	Sigma-Aldrich	98.0
Ethylene glycol	Sigma-Aldrich	98.0
Furfural	Sigma-Aldrich	99.0
Graphene oxide	Graphenea	GO water dispersion (0.4 wt %)
Hydrochloric acid	Sigma-Aldrich	37.0
Lithium chloride	Acros Organics	99.0
Methylene blue hydrate	TCI	70.0
Nafion (D-520 dispersion)	Alfa-Aesar	5% w/w in H_2O/1-propanol
Potassium hydroxide	Sigma-Aldrich	≥85.0, pellets
Potassium permanganate	Sigma-Aldrich	97.0
8-Quinolineboronic acid	Fluorochem	98.0
Ruthenium chloride	Fluorochem	98.0
Sodium hydroxide	Fluorochem	97.0, pellets
Tetrabutylammonium hexafluorophosphate	Sigma-Aldrich	≥99.0 (electrochemical analysis grade)
Thionyl chloride	Sigma-Aldrich	≥99.0
Triethylamine	Sigma-Aldrich	≥99.0

Solvents used in this work.

Solvent	Supplier	Purity (%)
Acetone	Fischer Scientific	>99.0
Acetonitrile	Sigma-Aldrich	>99.0
Chloroform	Sigma-Aldrich	≥99.8
1,4-Dioxane	Sigma-Aldrich	>99.9
Deuterium oxide	Deutero	>99.9
Ethanol	Sigma-Aldrich	≥99.0
Methanol	Sigma-Aldrich	>99.0
Water	Distilled in-house	-

2. Characterisations

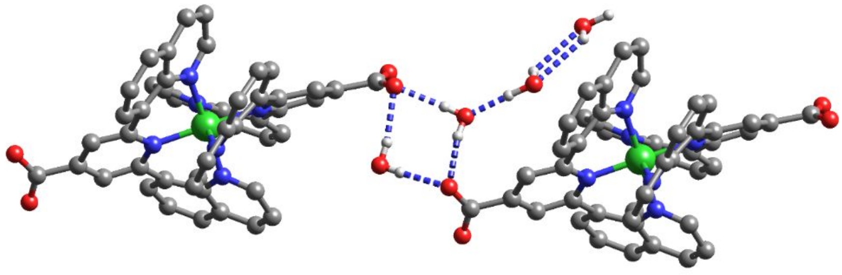

Figure S1. Crystal structure of [Ru(dqpCOO)$_2$].$_2H_2O$, hydrogens not involved in hydrogen bonding (blue dashed lines) are omitted for clarity. The picture was generated using the Oscail software package. Key: white – hydrogen, grey – carbon, blue – nitrogen, red – oxygen, green – ruthenium.

Table S1. Crystal structure data and structure refinement for [Ru(dqpCOO)$_2$].4H_2O

**Parameter**	**[Ru(dqpCOO)$_2$].4H_2O**
Empirical formula	$C_{24}H_{18}N_3O_4Ru_{0.50}$
Formula weight	462.95
Temperature	298.0(7) K
Wavelength	0.71073 Å
Crystal system	Monoclinic
Space group	C2/c
Unit cell dimensions	a = 15.7708(4) Å<br>b = 17.0565(4) Å<br>c = 14.3100(3) Å
Volume	3786.44(16) Å^3
Z	8
Density (calculated)	1.624 Mg/m^3
Absorption coefficient	0.485 mm^{-1}
F(000)	1896
Crystal size	0.50 x 0.40 x 0.20 mm^3
Theta range for data collection	3.551 to 29.264°.
Index ranges	-21<=h<=19, -23<=k<=23, -19<=l<=19
Reflections collected	16475
Independent reflections	4576 [R(int) = 0.0436]
Completeness to theta = 25.242°	99.7 %
Absorption correction	Semi-empirical from equivalents
Max. and min. transmission	1.00000 and 0.76913
Refinement method	Full-matrix least-squares on F^2
Data / restraints / parameters	4576 / 0 / 301
Goodness-of-fit on F^2	1.148
Final R indices [I>2σ(I)]	R1 = 0.0366, wR2 = 0.0906
R indices (all data)	R1 = 0.0449, wR2 = 0.0974
Extinction coefficient	n/a
Largest diff. peak and hole	1.260 and -0.734 e.Å^{-3}

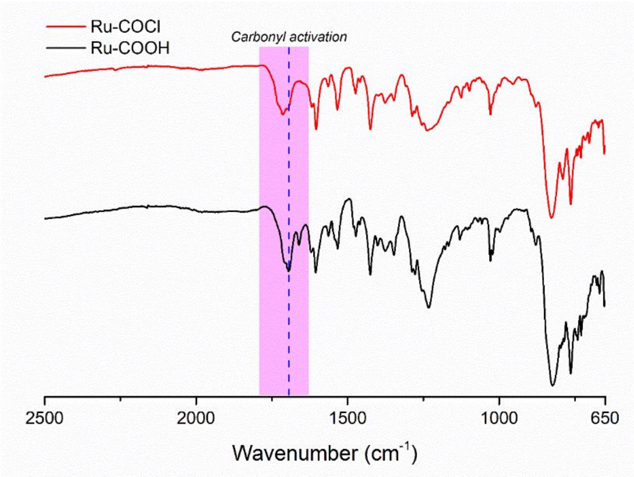

Figure S2. Infrared spectroscopy of $[Ru(tpyCOCl)_2]^{2+}$ (red) and $[Ru(tpyCOOH)_2]^{2+}$ (black).

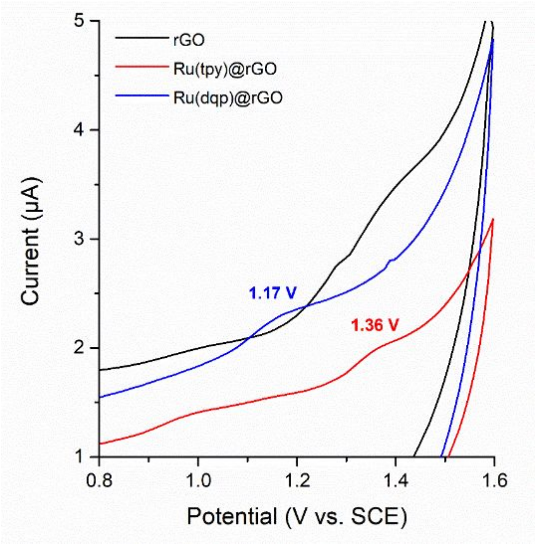

Figure S3. Overlayed cyclic voltammograms of rGO (black), Ru(tpy)@rGO (red) and Ru(dqp)@rGO (blue). All measurements were performed in N_2-bubbled CH_3CN at a scan rate of 0.1 V/s. GO-based materials were drop-cast onto glassy carbon electrodes *via* a Nafion/EtOH suspension. E^{Ox} values for the $Ru^{III/II}$ couple are indicated in their respective colours.

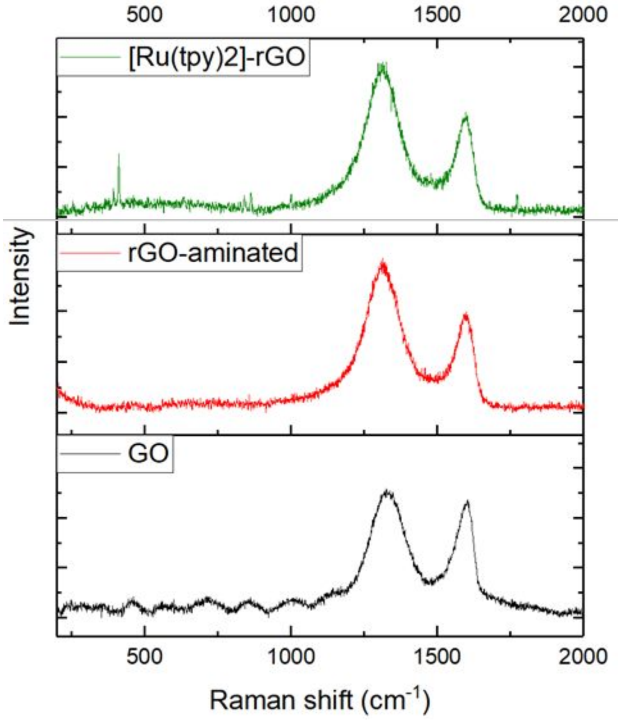

Figure S4. Resonance Raman spectra of GO (black), rGO (red) and Ru(tpy)@rGO (green) at λ_{exc} = 785 nm.

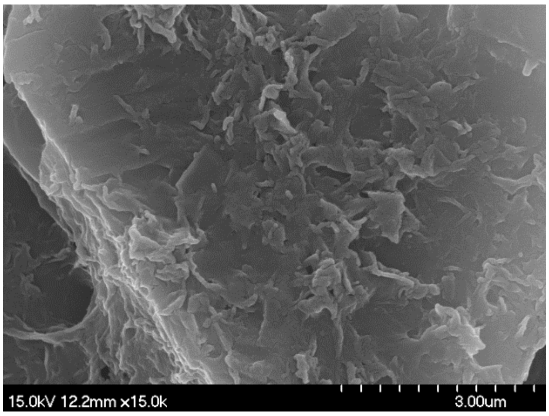

Figure S5. SEM image of rGO.

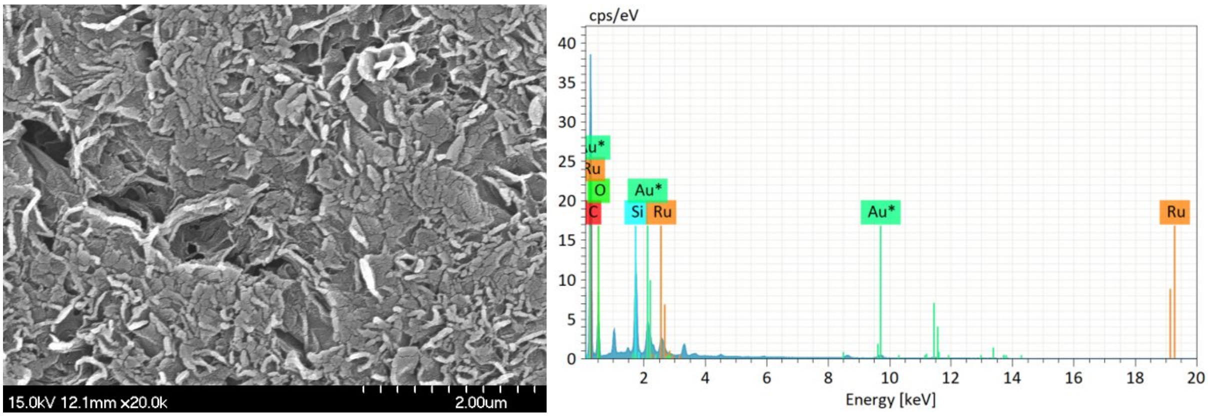

Figure S6. SEM image and corresponding EDX spectrum of Ru(tpy)@rGO.

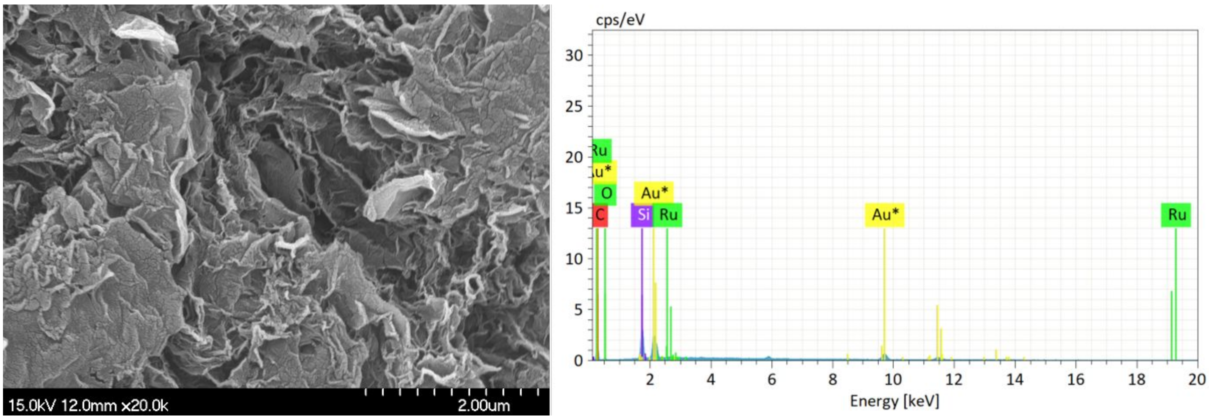

Figure S7. SEM image and EDX spectrum of Ru(dqp)@rGO.

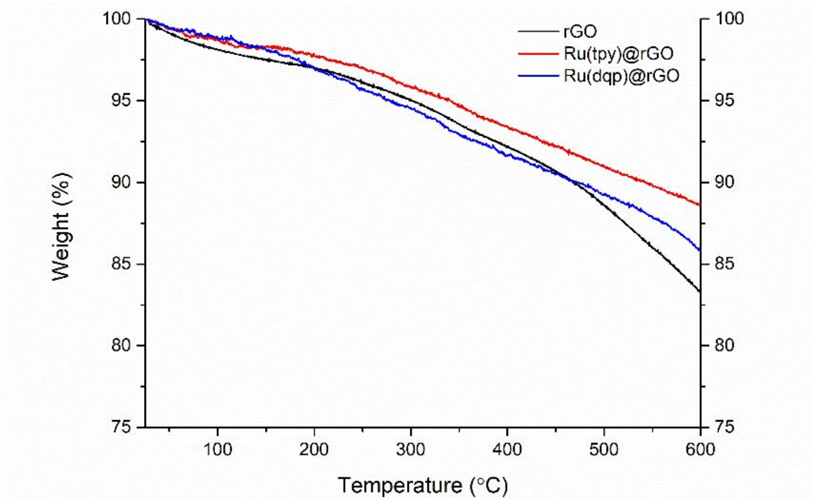

Figure S8. TGA of rGO (black), Ru(tpy)@rGO (red) and Ru(dqp)@rGO.

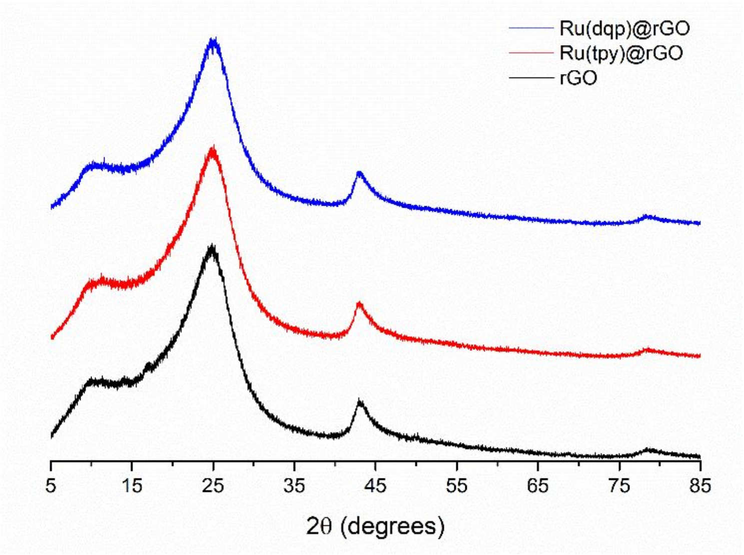

Figure S9. PXRD of rGO (black), Ru(tpy)@rGO (red) and Ru(dqp)@rGO (blue).

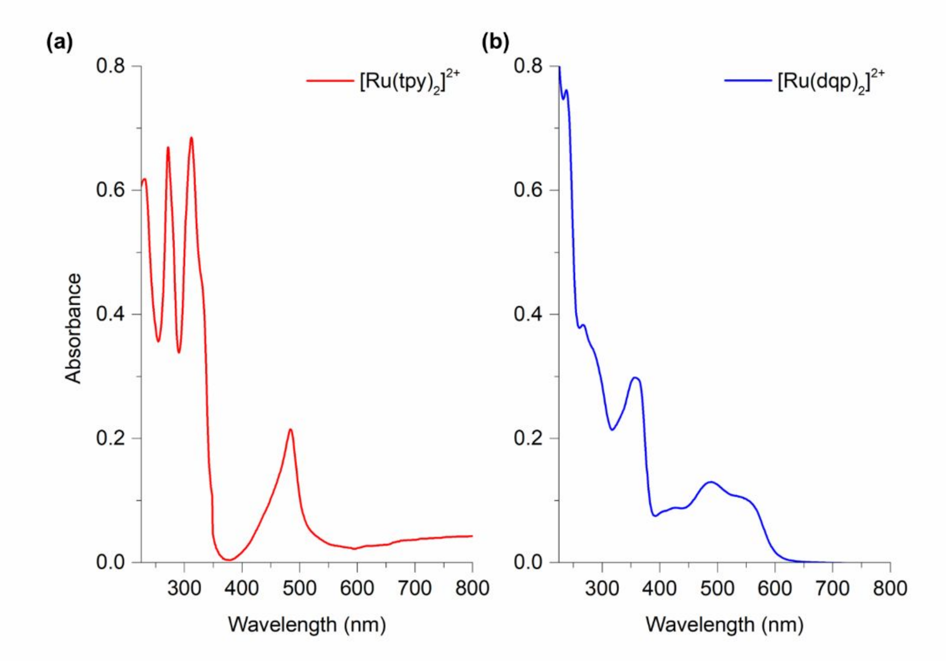

Figure S10. UV-Vis spectra of **(a)** $[Ru(tpyCOOH)_2]^{2+}$ and **(b)** $[Ru(dqpCOOH)_2]^{2+}$ both performed in dry CH_3CN.

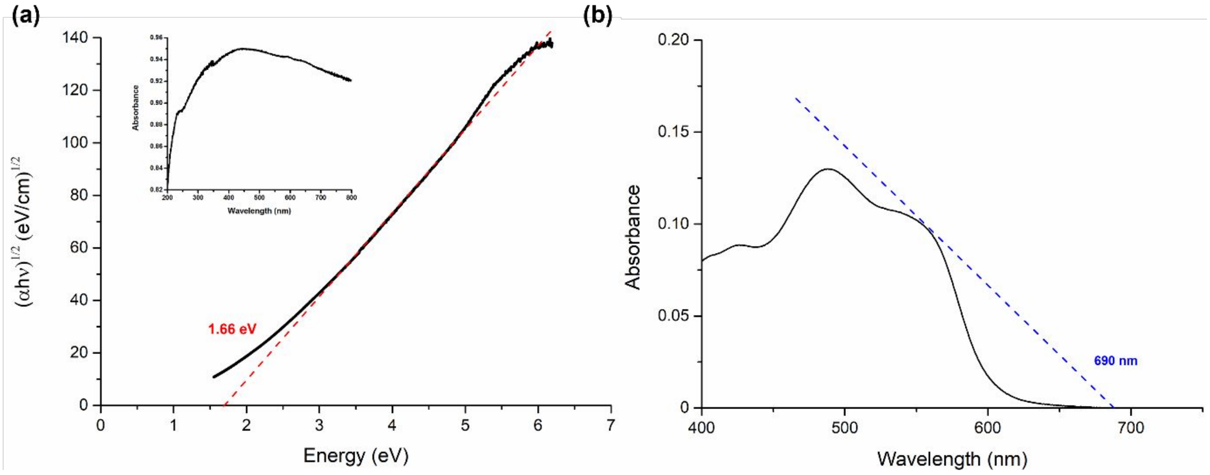

Figure S11. (**a**) Tauc plot of rGO, calculated from ss-UV-Vis (inset). Experimental E_g value highlighted. (**b**) Absorbance spectra of $[Ru(tpyCOOH)_2]^{2+}$, experimental excitation energy highlighted.

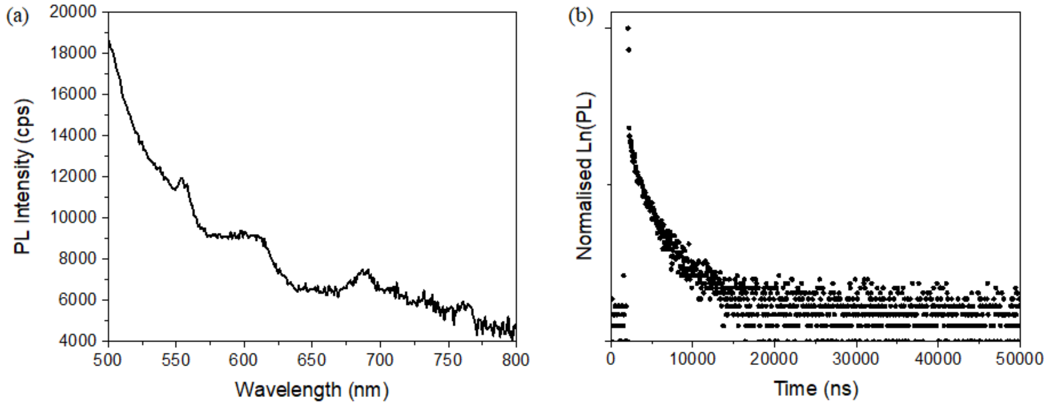

Figure S12. (**a**): Emission spectrum of Ru(dqp)@rGO, recorded in the solid state at $\lambda_{exc} = 480$ nm, with 10 nm slits. (**b**): Excited-state decay profile of Ru(dqp)@rGO, recorded at $\lambda_{em} = 690$ nm.

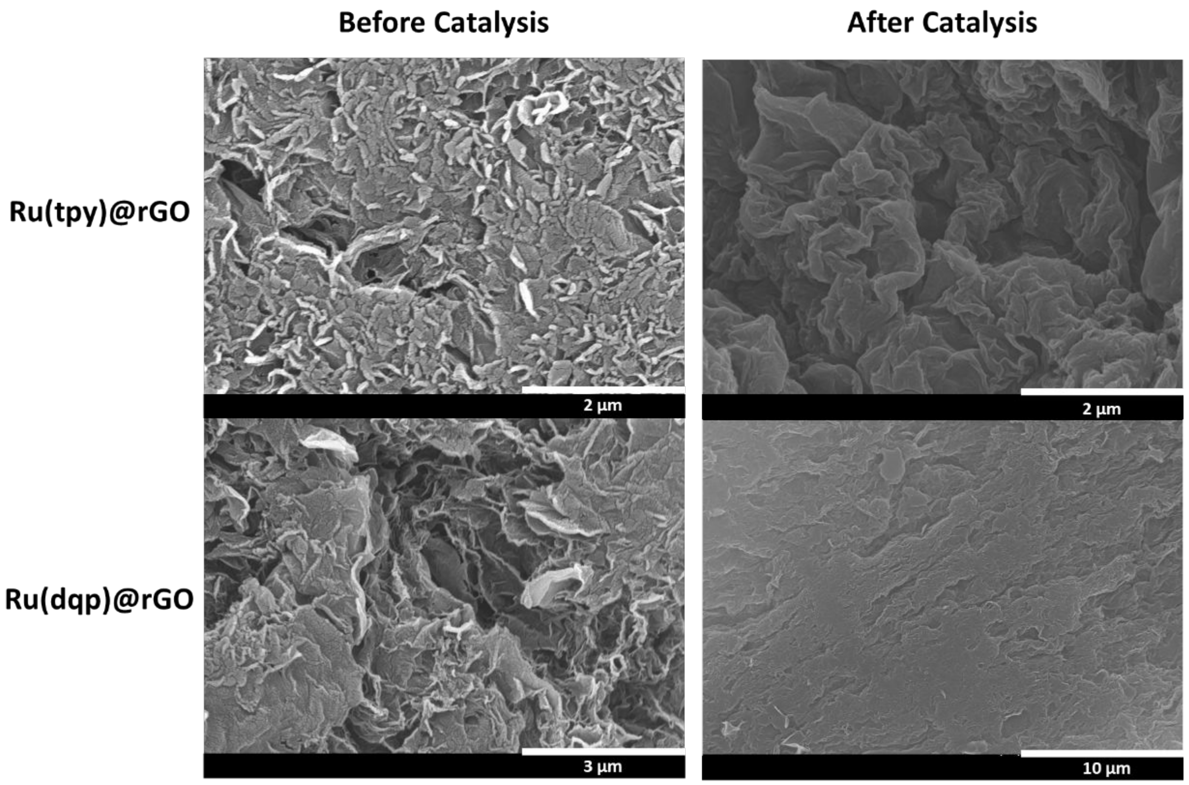

Figure S13. SEM images of before and after MB photodegradation studies.

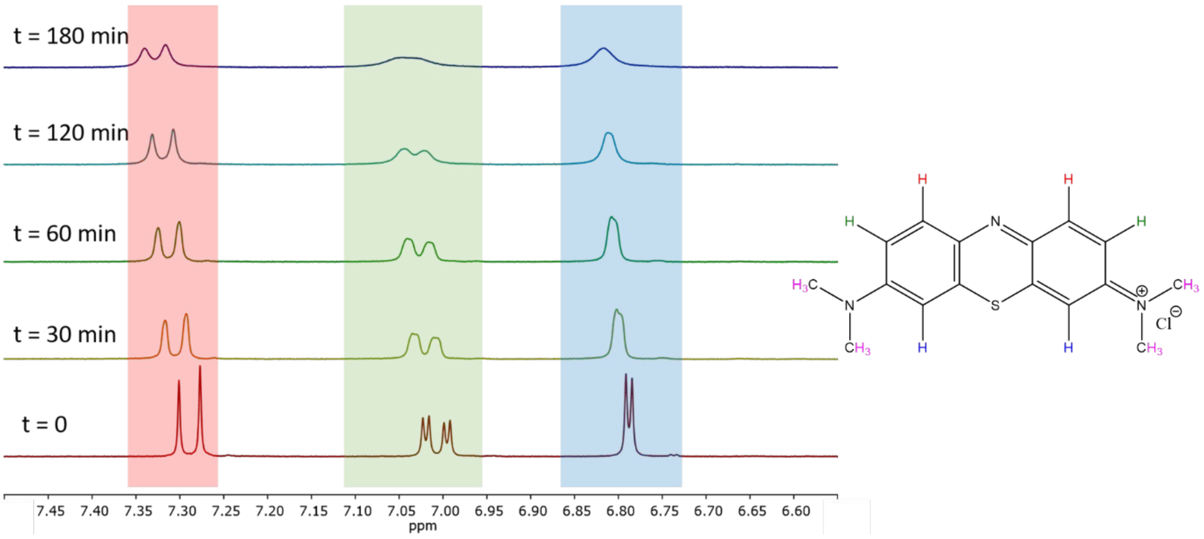

Figure S14. Aromatic region of the ^{1}H-NMR spectra of MB (2000 ppm) over the course of a 3 h photodegradation experiment using Ru(dqp)@rGO in D_2O. Highlighted regions corresponding to each type of aromatic proton.

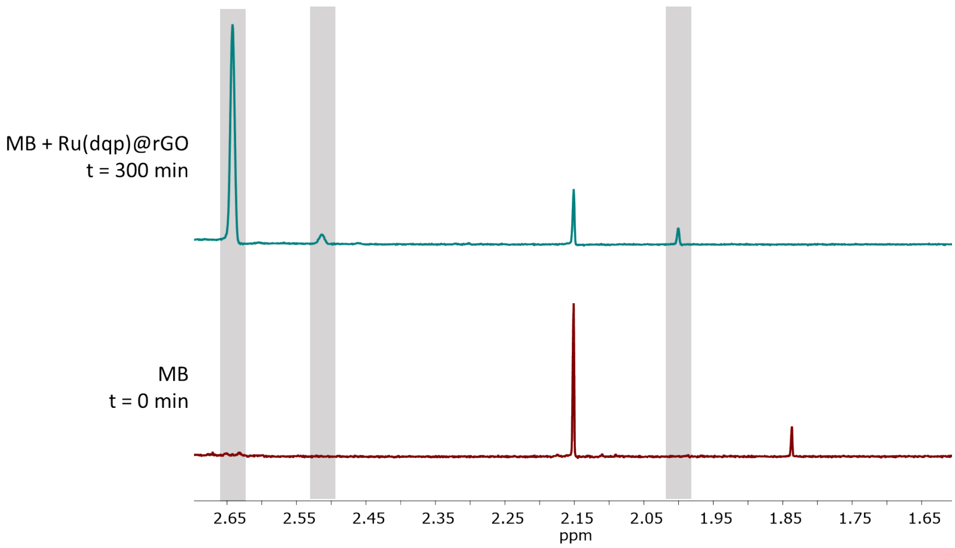

Figure S15: Aliphatic region of the ^{1}H-NMR spectra of MB (2000 ppm) pre-irradiation (burgundy) and MB post-irradiation using Ru(dqp)@rGO and visible light (green) in D_2O. Highlighted regions correspond to new peaks assigned to MB degradation products.

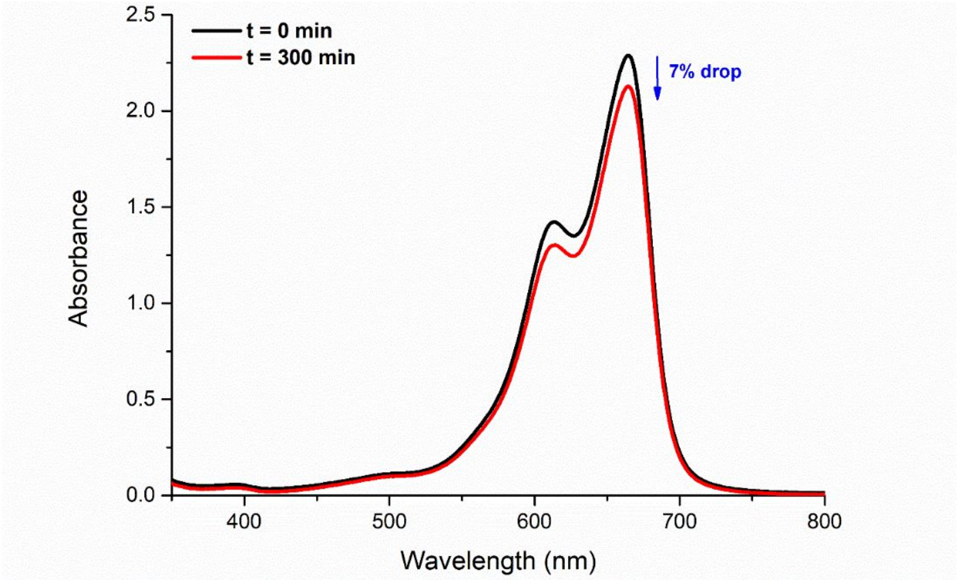

Figure S16: UV-Vis of MB (2000 ppm) pre-irradiation (black) and MB post-irradiation using Ru(dqp)@rGO under 1 sun LED (red). Solutions were diluted by a factor of x200 to obtain the spectra.
